# Hierarchical-Circular Model of Biological Memory: an integrative framework for pathogenesis and allostasis in neurodevelopmental disorders

**DOI:** 10.3389/fnbeh.2026.1779059

**Published:** 2026-03-23

**Authors:** Samuel Ruesga Mundo

**Affiliations:** High Specialty Medical Unit, Mexican Social Security Institute, Western National Medical Center, Guadalajara, Jalisco, Mexico

**Keywords:** allostatic load, biological memory, circular causality, developmental plasticity, neurodevelopmental disorders, PNEI network, systems theory

## Abstract

**Introduction:**

Predominant linear and brain-centric models inadequately explain the marked clinical heterogeneity and systemic origins of neurodevelopmental disorders (NDDs). A transformative, integrative framework is needed to capture their complex, non-linear pathogenesis.

**Methods:**

We propose a novel theoretical hypothesis—the Hierarchical-Circular Model of Biological Memory—developed through a critical synthesis of multidisciplinary evidence. Organized around the core principle “Signal → Plasticity → Stable State,” the model integrates five interacting levels: (1) morphogenetic/genetic, (2) epigenetic, (3) allostatic, (4) the psychoneuroendocrine-immune (PNEI) network, and (5) the interoceptive-neuronal level.

**Results:**

The framework posits that NDDs emerge from disrupted circular causality within biological adaptation systems. Early adverse signals (e.g., genetic risk, prenatal stress) become embedded via epigenetic programming and propagate bidirectionally, establishing a pathological stable state. This state is characterized by high allostatic load, PNEI network dysregulation, and a collapse of predictive interoceptive integration in the brain. The model introduces the constructs of “allostatic integrity” and “circular reserve” to explain individual differences in phenotypic expression and resilience.

**Discussion and conclusion:**

This model provides a falsifiable, systems-based paradigm that moves beyond descriptive synthesis. It generates specific predictions: (1) multi-level biomarker dyssynchrony will outperform single-level measures in prognostic stratification, and (2) interventions simultaneously targeting multiple system levels will be most effective. By bridging gene-environment interactions with brain network dysfunction, the framework guides future research toward multi-level biomarker discovery, personalized prevention, and multidimensional interventions, fundamentally redefining NDDs as disorders of circular biological adaptation.

## 1 Introduction

The rising prevalence of neurodevelopmental disorders (NDDs), including Autism Spectrum Disorder (ASD) and Attention-Deficit/Hyperactivity Disorder (ADHD), constitutes a major challenge for public health and neuroscience (Jubair, 2025). Despite extensive research, their profound clinical heterogeneity and complex etiology remain inadequately explained. Traditional approaches often isolate genetic risk, early environmental insults, or later brain circuit dysfunction, creating a fragmented understanding. The limited success of therapies targeting single mechanisms underscores the insufficiency of linear, reductionist models and highlights the urgent need for integrative frameworks that capture the dynamic, multi-system nature of NDD pathogenesis (Guzzetta et al., 2024).

It is well-established that NDDs originate from complex gene-environment interactions beginning *in utero*. Prenatal exposures, such as maternal stress or immune activation, can alter fetal brain programming, with effects moderated by the child's genotype (Ander et al., 2025; Mulder et al., 2025). These early experiences become biologically embedded via epigenetic mechanisms, such as DNA methylation, dysregulating genes critical for neurodevelopment and synaptic plasticity (Jubair, 2025; Meijer et al., 2025). However, these molecular imprints do not act in isolation. They initiate and are sustained by dysregulation across broader physiological systems.

A systems perspective is therefore essential. The cumulative physiological cost of adaptation, quantified as allostatic load, reflects how recurrent stress responses across development can dysregulate metabolic, immune, and neuroendocrine systems (Lupien et al., 2009). Crucially, the communication between these peripheral systems and the brain is orchestrated by the Psycho-Neuro-Endocrine-Immunological (PNEI) network, a master regulator of the body's response to internal and external demands (Santamaría-García et al., 2025). Disruption within this network creates feedforward loops, where peripheral inflammation and stress signals exacerbate central dysfunction, and vice-versa.

Ultimately, these cascading disruptions manifest in the brain as altered neural connectivity and a failure of predictive integration (Chen et al., 2021). Converging evidence identifies disrupted functional connectivity, particularly in thalamocortical and fronto-temporal networks, as a core feature of conditions like ASD (Lanciano et al., 2025; Karim and Rathore, 2025). This is intimately linked to impaired interoception—the brain's ability to sense, integrate, and predict bodily states—which is recognized as a transdiagnostic deficit across psychiatric and neurological disorders, including ASD (Yang et al., 2024; Zoltowski et al., 2025; Hazelton et al., 2025). The interoceptive network, centered on the anterior insula and cingulate cortex, may represent a final common pathway where systemic dysregulation translates into altered socio-cognitive and emotional processing (Santamaría-García et al., 2025; Zatorre et al., 2012).

Despite this knowledge, a critical theoretical gap persists. While influential frameworks—such as the Developmental Psychopathology perspective (focusing on equifinality/multifinality) and Allostatic Load theory (focusing on physiological wear and tear)—have advanced the field, none provide an integrative model that explicitly links and explains the recursive interactions across all relevant levels of analysis: from prenatal gene-environment interactions and epigenetic programming, through systemic physiological dysregulation (allostatic load/PNEI), to the ultimate collapse of brain network integration and interoceptive awareness. Current research often operates in silos, without a cohesive model to explain how these levels interact circularly across the lifespan to establish and maintain a pathological state.

**Figure 1:**
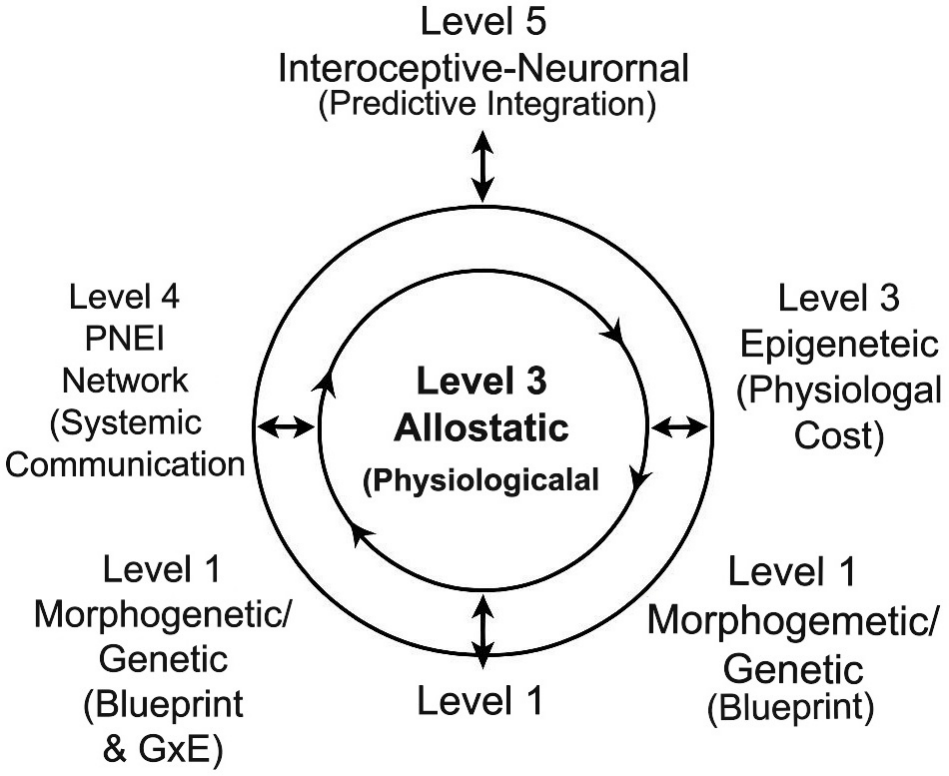
Hierarchical-Circular Model of Biological Memory. The schematic illustrates the five interconnected levels of the model, organized around the unifying principle “Signal → Plasticity → Stable State.” Arrows depict the bidirectional, circular flow of information and influence across levels, emphasizing the system's dynamic and integrated nature.

Here, we propose a novel theoretical hypothesis to bridge this gap: the Hierarchical-Circular Model of Biological Memory. We define biological memory in a broad, systemic sense (distinct from its narrower use in immunology) as the capacity of an organism's integrated systems to retain information from past experiences and stressors through stable plastic changes (epigenetic, physiological, network-based), thereby durably shaping future adaptive responses and vulnerability. Organized around the core, unifying principle of “Signal → Plasticity → Stable State,” our model integrates five interconnected levels of biological organization: (1) Morphogenetic/Genetic, (2) Epigenetic, (3) Allostatic, (4) the PNEI Network, and (5) the Interoceptive-Neuronal level (see Figure 1).

This manuscript establishes the theoretical foundations of this model. We demonstrate how it provides a coherent, mechanistic, and falsifiable explanation for the pathogenesis and heterogeneity of NDDs by synthesizing and integrating evidence across disciplines. The model posits that NDDs are not merely “brain disorders” but disorders of circular biological adaptation systems, where a perturbation at any level can propagate bidirectionally, culminating in a maladaptive yet structured stable state. By introducing novel constructs such as “allostatic integrity” and “circular reserve,” and by offering a roadmap for multi-level biomarker integration and multidimensional interventions, this framework aims to catalyze a paradigm shift from isolated, linear causality to a dynamic, systems-based understanding of neurodevelopmental vulnerability, resilience, and personalized intervention.

## 2 Materials and methods

### 2.1 Development of the theoretical framework and hypothesis

The Hierarchical-Circular Model of Biological Memory was developed as a novel theoretical hypothesis through an iterative, multi-phase process of critical synthesis and interdisciplinary integration, consistent with the standards for conceptual/theoretical articles. This process involved:

- **Phase 1: Critical Analysis of Limitations**. We systematically identified the explanatory shortcomings of prevailing linear and brain-centric models of NDD pathogenesis by analyzing paradoxical clinical and epidemiological findings, as well as the high failure rate of single-mechanism therapeutic trials.
- **Phase 2: Systems Mapping**. We conducted a comprehensive mapping of biological systems and constructs known to demonstrate reciprocal, non-linear interactions relevant to neurodevelopment and mental health across multiple temporal scales (prenatal, early-life, adult).
- **Phase 3: Framework Integration and Novelty Identification**. We integrated these mapped systems into a coherent, multi-level framework. Crucially, we explicitly defined its novel contributions by contrasting it with existing integrative frameworks (see Table 1 in the Discussion), emphasizing the unique integration of five specific levels, the “Signal → Plasticity → Stable State” principle, and the emphasis on circular causality over linear or simply bidirectional models.

**Table 1:** Comparison with existing integrative frameworks.

**Feature**	**Developmental psychopathology**	**Allostatic load theory**	**Hierarchical-circular model (proposed)**
Core focus	Pathways to disorder (equifinality/multifinality)	Physiological cost of chronic stress	Circular causality across 5 system levels
Temporal dynamics	Lifespan development	Cumulative wear and tear	“Signal → plasticity → stable state” loop
Levels integrated	Psychological, environmental, biological (broad)	Physiological systems	5 specified tiers (morphogenetic to neuronal)
Key construct	Risk/resilience, developmental trajectories	Allostatic load/overload	Allostatic integrity, circular reserve
Clinical implication	Early intervention, multi-contextual assessment	Reduce chronic stress	Break pathological cycles via multi-level interventions

### 2.2 Literature synthesis strategy

To ground the hypothesis in contemporary evidence, we conducted a targeted, narrative synthesis of multidisciplinary literature published between 2010 and early 2025. This period captures the modern era of systems biology and biomarker research in neurodevelopment. Searches were performed in PubMed, Web of Science, and Scopus using key term combinations related to the model's core constructs (e.g., “allostatic load” AND neurodevelopment, “PNEI network,” “interoception” AND autism, “epigenetics” AND synaptic plasticity).

**Guiding Principles for Evidence Selection:** Inclusion was guided by the need to support a plausible and coherent theoretical argument, prioritizing:

1. Longitudinal human studies tracking developmental trajectories or cross-system interactions over time.
2. Mechanistic investigations (human or animal) elucidating pathways between at least two systems (e.g., stress → inflammation → neural function).
3. Seminal reviews and high-impact empirical studies providing robust evidence for interactions within or between the five levels of the proposed model.
4. Research demonstrating non-linear, recursive, or bidirectional relationships between variables, as these are central to the model's logic.

We explicitly acknowledge that this is not a systematic review or meta-analysis. The synthesis is selective and interpretative, aimed at constructing a logically sound theoretical model rather than providing an exhaustive summary of all evidence. Consequently, formal risk-of-bias assessments (e.g., NOS, SYRCLE) and quantitative evidence grading (e.g., CEBM) were not performed, as they are not standard for theoretical articles of this nature. The model's validity is judged by its explanatory power, internal coherence, and ability to generate testable predictions.

### 2.3 Integration of cross-disciplinary evidence and novel construct development

A core methodological challenge was integrating evidence from disparate fields (genetics, epigenetics, stress physiology, immunology, systems neuroscience). We employed the following strategies:

- Convergence Analysis: Identifying points where findings from different methodologies (e.g., neuroimaging, biomarker assays, behavioral tasks) converged on a common mechanism or dysfunction (e.g., interoceptive impairment in ASD).
- Temporal and Causal Inference: Using evidence from longitudinal and intervention studies to infer plausible sequences of disruption and bidirectional influences across the lifespan.
- Construct Operationalization: Developing initial, testable definitions for the novel constructs introduced by the model (“allostatic integrity,” “circular reserve”) by proposing specific, multi-system biomarker profiles and dynamic response patterns that could be measured in future research.

### 2.4 Hypothesis generation and criteria for theoretical utility

The ultimate aim of this theoretical work is to generate a falsifiable hypothesis. We evaluated the model's utility based on its capacity to:

1. Provide a Unifying Explanation: Offer a coherent narrative for clinical observations poorly explained by existing models (e.g., heterogeneity, treatment resistance).
2. Generate Novel, Testable Predictions: Formulate specific, discriminative hypotheses that can be empirically tested (detailed in the Discussion).
3. Identify Translational Novelty: Propose new intervention targets and strategies that differ from conventional single-target approaches by focusing on restoring multi-level synchrony.
4. Serve as an Integrative Framework: Provide a structured scaffold for designing future studies that collect and analyze multi-level data (genomic, epigenetic, physiological, neural, behavioral).

## 3 Results

### 3.1 The hierarchical-circular model: evidence and interactions across levels

#### 3.1.1 Level 1: morphogenetic/genetic the blueprint and its conditional expression

The foundational level establishes a blueprint for development and a range of potential trajectories. Evidence confirms that prenatal signals, such as maternal stress or immune activation, can durably alter brain development, but crucially, these effects are moderated by the child's genotype via gene-environment interactions (GxE) (Ander et al., 2025; Mulder et al., 2025). For instance, the impact of prenatal stress on DNA methylation in genes critical for neurodevelopment (e.g., CHD2, ORC5) is significantly modulated by fetal genetic variants (Alemany et al., 2021). This demonstrates the initial “Signal → Plasticity” step, where an adverse environmental signal interacts with a genetic substrate to produce a specific molecular change, setting a trajectory toward increased system vulnerability. Circular Connection: This initial programming creates a molecular substrate that influences all higher levels. For example, GxE-induced alterations can preconfigure the responsiveness of stress and immune pathways, thereby shaping the future function of the PNEI network (Level 4) from the outset (Tanner and Özkaya, 2021).

#### 3.1.2 Level 2: epigenetic—the molecular memory of experience

This level serves as the primary interface for encoding early experiences into stable changes in gene regulation. Environmental exposures (stress, nutrition, toxins) leave lasting epigenetic marks (e.g., DNA methylation) that dysregulate genes essential for neurogenesis, synaptic plasticity, and immune function (Jubair, 2025; Meijer et al., 2025). Critically, these marks are cell-type specific. In ADHD, distinct DNA methylation patterns are found in glutamatergic neurons, GABAergic neurons, and microglia within the same brain regions, highlighting the cellular precision of this “molecular memory” and its potential for circuit-specific effects (Vidal et al., 2025). Circular Connection: Epigenetic modifications durably modulate systems central to adaptation. For example, methylation of the glucocorticoid receptor gene (NR3C1) can alter HPA axis reactivity, directly contributing to allostatic load (Level 3) (Tyrka et al., 2016). This persistent physiological state acts as a new signal, potentially exacerbating PNEI dysregulation (Level 4) and distorting interoceptive input (Level 5).

#### 3.1.3 Level 3: allostatic—the physiological cost of adaptation

Allostatic load quantifies the cumulative physiological wear and tear from recurrent stress responses. In neurodevelopment, early adversity leads to dysregulation across metabolic, immune, cardiovascular, and neuroendocrine systems (Lupien et al., 2009; Deighton et al., 2021). This is not a passive endpoint but an active driver of pathology. The related concept of “allostatic-interoceptive overload” describes a failure in predictive bodily regulation, a transdiagnostic mechanism in psychiatric and neurological disorders (Santamaría-García et al., 2025). High allostatic load in childhood is a physiological memory of adversity that predicts subsequent cognitive and emotional difficulties (Juster et al., 2010). Circular Connection: Elevated allostatic load is both an outcome and a perpetuating factor. High cortisol and inflammatory cytokines can induce new epigenetic changes (Level 2) (Davies et al., 2019), disrupt PNEI network communication (Level 4), and impair prefrontal and limbic function (Level 5), thereby consolidating the pathological “Stable State.”

#### 3.1.4 Level 4: the PNEI network—the central conduit of systemic communication

The Psychoneuroendocrine-Immunological (PNEI) network is the master bidirectional mediator between brain and body. Its dysregulation is central to allostatic-interoceptive overload (Santamaría-García et al., 2025). Mechanistic evidence shows specific pathways: risk alleles (e.g., APOE4) can alter microglial signaling, leading to neuronal hyperexcitability and metabolic stress, directly linking genetic risk (Level 1) to neuroimmune dysfunction and neuronal impairment (Level 5) (Verduzco Espinoza et al., 2025). Furthermore, cytokines may affect brain function not only via humoral pathways but also via neural routes (e.g., vagus nerve), highlighting the complex, multi-route communication within the PNEI network (Tyagi and Bartley, 2025). Circular Connection: The PNEI network is the principal hub for circular flow. It translates signals from Levels 1–3 into endocrine/immune outputs that further increase allostatic load (Level 3). Simultaneously, its state (e.g., peripheral inflammation) shapes the sensory input that reaches interoceptive brain regions (Level 5), directly affecting the brain's model of the body.

#### 3.1.5 Level 5: interoceptive-neuronal—predictive integration and its collapse

The highest level involves the brain's integration of bodily signals for self-regulation and social cognition. In NDDs, this system shows impairment at multiple scales. Functionally, reduced connectivity between the anterior insula and anterior cingulate cortex is linked to autistic traits (Yang et al., 2024). At the network level, individuals with ASD show atypical mesoscopic patterns, including thalamic hyperconnectivity and altered frontal-occipital connectivity, reconciling previous disparate findings (Lanciano et al., 2025; Karim and Rathore, 2025). Structurally, alterations in cortical thickness and asymmetry in salience and default mode networks are associated with comorbid internalizing/externalizing symptoms in developmental populations (Olson et al., 2021; Wild, 2012). Dynamically, altered intrinsic neural timescales in interoceptive regions suggest less efficient temporal processing in conditions like ASD (Serdarevic et al., 2017). This multi-scale dysfunction represents the ultimate failure in predictive integration. Circular Connection: The failure at Level 5 is both consequence and cause. Impaired interoception leads to maladaptive behavioral and emotional responses, increasing psychological stress. This stress signal feeds back, exacerbating PNEI dysregulation (Level 4) and allostatic load (Level 3), thus closing the pathogenic loop.

#### 3.1.6 Synthesis: the model as a unifying explanatory framework

The circular architecture provides a coherent explanation for key challenges in the field. Clinical heterogeneity within ASD or ADHD can be reinterpreted as different configurations of “allostatic integrity” within the system. An individual with high genetic/epigenetic load (Levels 1–2) but strong PNEI regulation (Level 4) and preserved interoception (Level 5) may exhibit a milder phenotype due to high “circular reserve.” The model also explains intervention resistance: a behavioral therapy may fail if high allostatic load (Level 3) or inflammation (Level 4) maintains the pathological cycle. It predicts that interventions simultaneously targeting multiple levels will be most effective in restoring adaptive circularity.

## 4 Discussion

### 4.1 Theoretical integration, novelty, and differentiation from existing frameworks

The Hierarchical-Circular Model constitutes a novel theoretical hypothesis that advances beyond existing integrative frameworks. While Developmental Psychopathology excellently describes equifinality/multifinality, and Allostatic Load Theory quantifies physiological wear, our model uniquely (1) integrates five specific levels of analysis into a single schematic, (2) proposes the “Signal → Plasticity → Stable State” as a universal operating principle across these levels, and (3) emphasizes circular causality as the engine that establishes and maintains a maladaptive yet structured stable state (see Table 1 for comparison). This allows us to specify testable mechanisms of cross-level propagation, such as how an epigenetic mark influences a network dysfunction.

### 4.2 Reconciling heterogeneity and informing intervention

The model powerfully explains clinical heterogeneity through the constructs of allostatic integrity (the system's current functional synchrony) and circular reserve (its capacity to maintain function despite perturbation). This clarifies why biomarkers often correlate poorly with symptoms: an individual's phenotype depends on the dynamic configuration of all levels, not a single one. Consequently, the model predicts that unimodal interventions will show inconsistent efficacy, as they fail to address the sustaining circular dynamics. It argues compellingly for multidomain, personalized interventions designed to “break” key pathological loops (e.g., combining anti-inflammatory support with interoceptive training).

### 4.3 Disorder-specific considerations and comorbidity

Although the model is transdiagnostic, it provides a lens to hypothesize disorder-specific patterns. In ASD, the primary entry point may often involve Levels 1–2 (genetic/epigenetic programs for synaptic and social brain development), with pronounced downstream effects on Level 5 (interoceptive-social brain integration). In ADHD, dysregulation might more prominently involve Levels 3–4 (allostatic and catecholaminergic systems), impacting fronto-striatal circuits. Comorbidities (e.g., anxiety in ASD) can be understood as the spreading of dysregulation to related circuits (e.g., amygdala), shaped by an individual's specific “circular” vulnerability (Beauchaine and Cicchetti, 2019; Cicchetti and Rogosch, 2002).

### 4.4 Falsifiable predictions and future research directions

A core strength of the model is its generation of specific, testable predictions:

1. **Prediction 1 (Biomarker Synchronicity):** In longitudinal cohorts, dyssynchrony profiles (mismatches between biomarker levels across different tiers, e.g., high inflammation with low cortisol) will predict symptom progression and comorbidity better than any single biomarker.
2. **Prediction 2 (Intervention Efficacy):** Multi-level interventions (e.g., targeting sleep/nutrition [Levels 3/4] + parent-mediated social coaching [Level 4/5]) will demonstrate superior and more sustained outcomes compared to single-domain treatments, particularly in measures of “circular flow” (e.g., heart-rate variability-brain connectivity coupling).
3. **Prediction 3 (Computational Modeling):** Agent-based or dynamical systems models implementing the proposed circular interactions will identify key leverage points (e.g., interoceptive accuracy) whose modulation *in silico* leads to the most efficient collapse of the pathological stable state.

### 4.5 Operationalizing translational constructs: toward an “allostatic integrity index”

To bridge theory and practice, we propose operationalizing “allostatic integrity” via a composite index. A hypothetical dashboard could include: (a) Inverse Load Metrics (high HRV, steep cortisol slope), (b) Inflammatory Resilience (high IL-10/IL-6 ratio), (c) Brain-Body Coupling (heart-evoked potential amplitude), and (d) Multi-System Synchrony (correlation strength between epigenetic age and physiological stress markers). “Circular reserve” could be assessed via the system's robustness and speed of recovery in a multi-system challenge paradigm.

### 4.6 Limitations and theoretical considerations

We acknowledge limitations. Empirically testing full circular dynamics requires unprecedented multi-level longitudinal data. The model is a heuristic; the precise weight and entry point of dysfunction will vary by individual and disorder subtype. It currently focuses on biological systems, though fully incorporating broader social and environmental “signals” (e.g., peer relationships, poverty) is essential for a complete picture (Rasheed and Jain, 2025; Zucker and Samson, 2023).

### 4.7 Conclusion of the discussion

This model reframes NDDs as dynamic disorders of circular biological adaptation. By integrating disparate evidence into a falsifiable hypothesis, it moves the field from a focus on static risk factors to the analysis of dysfunctional system dynamics. It provides a roadmap for a new generation of research and holistic clinical practice aimed at measuring and restoring allostatic integrity.

## 5 Conclusion

The Hierarchical-Circular Model of Biological Memory offers a transformative, systems-based paradigm for understanding neurodevelopmental disorders (NDDs). It posits that conditions like ASD and ADHD are best conceptualized not as linear outcomes of isolated deficits, but as emergent properties of a disrupted circular flow of information across genetic, epigenetic, physiological, network, and neural levels.

The primary contribution of this model is its capacity to integrate mechanistic evidence into a coherent, falsifiable narrative that explains heterogeneity, comorbidity, and treatment resistance. It introduces critical novel constructs allostatic integrity and circular reserve that shift the focus from isolated pathology to system-wide function and resilience.

While theoretical, the model is expressly designed to generate empirical research. It directs the field toward multi-level longitudinal studies, multidomain intervention trials, and computational modeling, all aimed at measuring and modulating the system's circular dynamics. Its translational imperative is clear: to advance from symptom-based diagnosis toward personalized, preventive strategies hat assess and support the integrity of the entire adaptive system from the beginning of life.

In conclusion, this framework provides the necessary theoretical scaffolding to advance neurodevelopment research into an era that embraces complexity, aiming not merely to classify symptoms but to understand and support the dynamic processes of healthy and altered adaptation.

## Data Availability

The original contributions presented in the study are included in the article/supplementary material, further inquiries can be directed to the corresponding author.
